# Factors associated with unreported tuberculosis cases in Spanish hospitals

**DOI:** 10.1186/s12879-015-1047-0

**Published:** 2015-07-29

**Authors:** Concepción Morales-García, Teresa Rodrigo, Marta M. García-Clemente, Ana Muñoz, Pilar Bermúdez, Francisco Casas, María Somoza, Celia Milá, Antón Penas, Carmen Hidalgo, Martí Casals, Joan A. Caylá, Working Group on Under-reporting of Tuberculosis in Spain

**Affiliations:** Programa Integrado de Investigación en Tuberculosis (PII-TB) de la Sociedad Española de Neumología y Cirugía Torácica (SEPAR), Barcelona, Spain; Hospital Universitario Virgen de las Nieves de Granada, Granada, Spain; Fundación Respira de la SEPAR, Barcelona, Spain; Hospital Central de Asturias de Oviedo, Oviedo, Spain; Hospital Universitario Carlos Haya de Málaga, Malaga, Spain; Hospital Universitario San Cecilio de Granada, Granada, Spain; Consorcio Sanitario de Tarrasa, Barcelona, Spain; Unitat de Prevenció i Control de Tuberculosis de Barcelona, Barcelona, Spain; Hospital Xeral-Calde de Lugo, Lugo, Spain; Agencia de Salud Publica, Barcelona, Spain; CIBER de Epidemiologia y Salud Pública (CIBERESP), Barcelona, Spain

**Keywords:** Notifications, Under-reporting, Reporting, Tuberculosis, Spain

## Abstract

**Background:**

Under-reporting of tuberculosis (TB) cases complicates disease control, hinders contact tracing and alters the accuracy of epidemiological data, including disease burden. The objective of the present study is to evaluate the proportion of unreported TB cases in Spanish healthcare facilities and to identify the associated factors.

**Methods:**

A multi-center retrospective study design was employed. The study included TB cases diagnosed in 16 facilities during 2011–2012. These cases were compared to those reported to the corresponding public health departments. Demographic, microbiological and clinical data were analyzed to determine the factors associated with unreported cases. Associated factors were analyzed on a bivariate level using the x^2^ test and on a multivariate level using a logistic regression. *Odds ratios* (OR) and 95 % confidence intervals (CI) were calculated.

**Results:**

Of the 592 TB cases included in the study, 85 (14.4 %) were not reported. The percentage of unreported cases per healthcare center ranged from 0–45.2 %. The following variables were associated to under-reporting at a multivariate level: smear-negative TB (OR = 1.87; CI:1.07-3.28), extrapulmonary disease (OR = 2.07; CI:1.05-4.09) and retired patients (OR = 3.04; CI:1.29-7.18). A nurse case manager was present in all of the centers with 100 % reporting. The percentage of reported cases among the smear-positive cases was 9.4 % and 19.4 % (p = 0.001) among the rest of the study population. Smear-positive TB was no associated to under-reporting.

**Conclusions:**

It is important that TB Control Programs encourage thorough case reporting to improve disease control, contact tracing and accuracy of epidemiological data. The help from a TB nurse case manager could improve the rate of under-reporting.

**Electronic supplementary material:**

The online version of this article (doi:10.1186/s12879-015-1047-0) contains supplementary material, which is available to authorized users.

## Background

Tuberculosis (TB) continues to be an important public health problem worldwide. In 2013, 9 million people developed TB and 1.5 million people died from the disease [1]. An estimated three million people with TB in 2012, one third of the total cases, were not reported to a national surveillance system [1]. Prevention and control requires quick and systematic reporting of new TB cases to surveillance centers to ensure treatment compliance and to facilitate contact tracing.

The incidence of reported TB cases in the European Union countries is 13.5 per 100,000 inhabitants [2] and is predominant among vulnerable populations [3]. Spain is considered a country of low TB incidence, with a rate of 14.7 cases per 100,000 in 2012 [2]. However, the distribution between different regions, or autonomous communities, is not equal, and ranges between 8 and 29 cases per 100,000 inhabitants [4].

The low TB incidence observed in Spain during recent years could be not only a result of disease control, but could also be a reflection of missed diagnoses or under-reporting [5, 6]. The potentially missed diagnoses or unreported cases would affect TB incidence in the country. This has been previously observed and published in other countries [6–12], but not well-studied in Spain. Data published on rate of unreported cases in Spain is limited to only one region [13–15], and was estimated at around 20 % [13–16]. In Galicia and Barcelona, two areas with effective TB control programs, the TB incidence is higher than the national average. This may be also due to under-reporting in other parts of Spain.

Identifying factors that are associated with under-reporting will allow us to target areas in need of better TB diagnosing and reporting, to in turn improve disease control. The objective of the present study is to describe the extent of unreported TB cases from healthcare facilities in various regions in Spain, and to identify the factors associated with unreported cases.

## Methods

### Study design

**Fig. 1:**
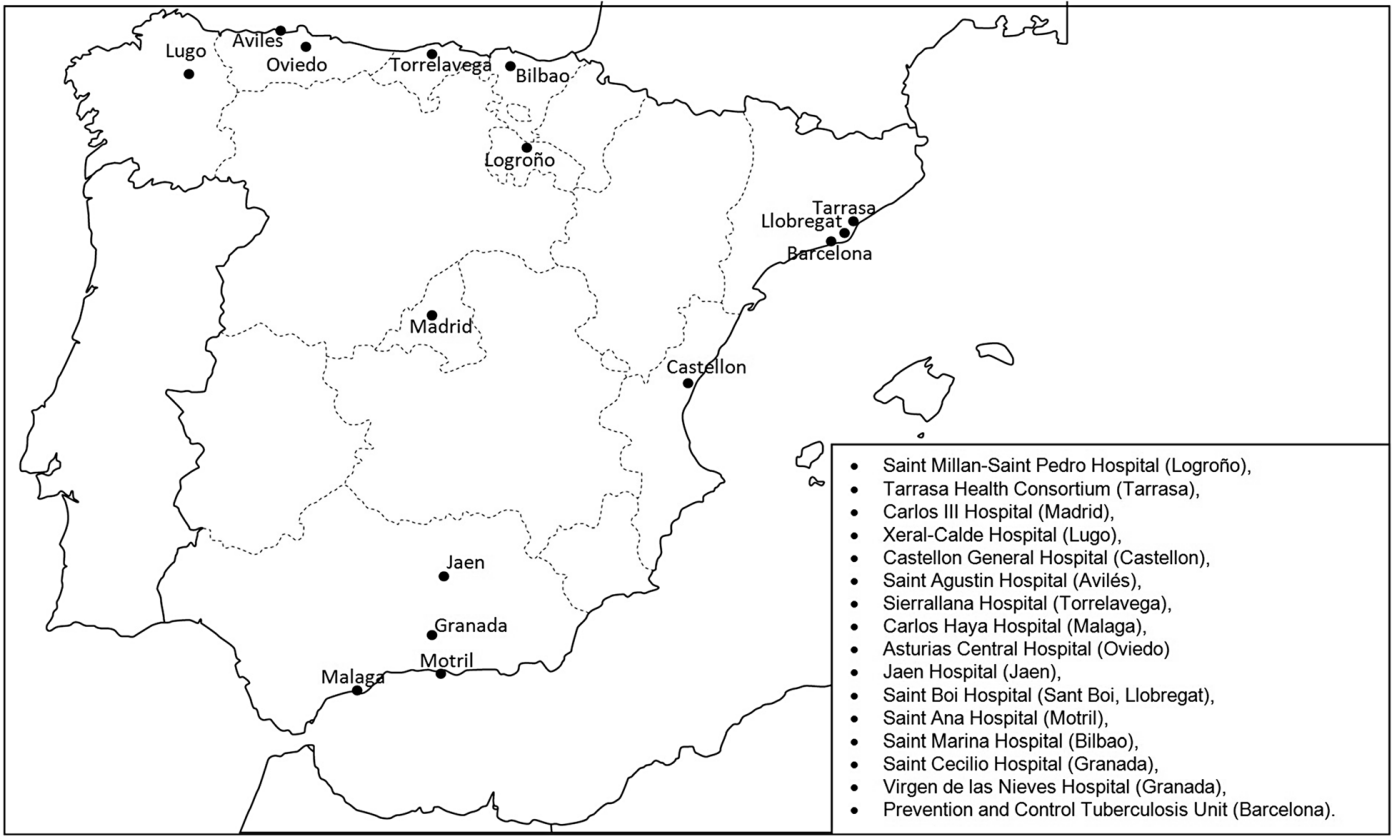
Geographical location of the participating healthcare centers in the study

This is a multi-center, retrospective study on a cohort of TB cases diagnosed in 16 hospitals in Spain (Fig. 1) from January 1^st^, 2011 to December 31^st^, 2012. The study includes TB cases detected by microbiological, pathological and clinical records of each healthcare facility, which were then compared to cases registered by healthcare facilities at their corresponding public health departments, including the Public Health Department of Andalusia, Asturias, Catalonia, Cantabria, Galicia, The Rioja, The Basque Country, Valencia and Madrid. Each case was classified as reported or not reported.

### Case definitions and data collection

Clinical, microbiological, and pathological documents were obtained from each healthcare facility, in both electronic and paper form. The following criteria were used for pulmonary and extrapulmonary TB diagnosis: microbiological confirmation of *Mycobacterium tuberculosis complex*, pathology report compatible with TB (ie caseous granulomas by biopsy), or absence of microbiological confirmation but medically-deemed active TB by clinical and radiological findings. TB cases per healthcare facility records were linked to a list of TB cases provided by the corresponding public health department.

Unreported case was defined as a case that was detected in hospital records but not present in the TB registry of the corresponding public health department.

TB cases were classified as smear-positive, smear-negative or extrapulmonary, according to WHO criteria [17].

Clinical and epidemiological data was collected from patient records and registries, and stored in a database with electronic access using identifying information with a password for each of the study investigators.

The following variables were studied: socio-demographic data (age, sex, country of origin, employment, living situation, site of diagnosis and toxic habits), clinical data (HIV co-infection, history of previous TB treatment, disease involvement and radiographic findings), and microbiological data (smear, culture and anti-TB drug sensitivity results).

### Ethics

The study was performed in accordance with the requirements stipulated in the Declaration of Helsinki (Tokyo revision, October 2004) and the Spanish Data Protection Act of 15/1999. The study was approved by the Independent Ethics Committees of the participating healthcare facilities (see Additional file 1).

### Statistical analysis

Reported TB cases were classified as “0” and unreported cases as “1.” The proportion of total unreported cases and proportion of unreported cases by healthcare facility were calculated. Absolute and relative frequencies were calculated for each variable and factors associated with unreported cases were analyzed on a bivariate level using the x^2^ test. A multivariate logistic regression model was constructed with the variables significant at the bivariate level, using manual stepwise selection to consider the factors with a p < 0.05 on a bivariate level. All variables without the presence of colinearity were included in the final model and interaction of covariates was evaluated.

Odds ratio (OR) and corresponding 95 % confidence intervals (CI) were calculated, and goodness of fit was tested using the Hosmer and Lemeshow test. P < 0.05 was considered statistically significant. IBM SPSS Statistics version 19.0 (SPSS Inc, Chicago, IL, USA) was used to perform all statistic analyses.

## Results

Of the 592 TB cases diagnosed between 2011–12 at the 16 participating healthcare facilities (Table 1), 85 cases (14.4 %) were not identified in the public health department registries. This proportion ranged from 0 to 45.2 % according to healthcare facility. One hundred percent of the TB cases from 5 healthcare facilities were reported to the corresponding public health department (all of which have a nurse case manager). The average of unreported cases was 20.7 % among the other 11 healthcare facilities. Microbiological confirmation was present for 509 cases (86 %).

**Table 1:** Distribution of the diagnosis and reported of tuberculosis according to healthcare facilities

Healthcare facility	Number of tuberculosis cases		Total
	Reported	Unreported	(N / %)
	(N / %)	(N / %)	
Saint Millan-Saint Pedro Hospital	21	10	31
	67.7 %	32.3 %	100.0 %
Tarrasa Health Consortium	38	0	38
	100.0 %	0.0 %	100.0 %
Carlos III Hospital	18	7	25
	72.0 %	28.0 %	100.0 %
Xeral-Calde Hospital	29	5	34
	85.3 %	14.7 %	100.0 %
Castellon General Hospital	24	3	27
	88.9 %	11.1 %	100.0 %
Saint Agustin Hospital	17	14	31
	54.8 %	45.2 %	100.0 %
Sierrallana Hospital	27	1	28
	96.4 %	3.6 %	100.0 %
Carlos Haya Hospital	69	0	69
	100.0 %	0.0 %	100.0 %
Asturias Central Hospital	46	24	70
	65.7 %	34.3 %	100.0 %
Jaen Hospital	11	1	12
	91.7 %	8.3 %	100.0 %
Saint Boi Hospital	21	0	21
	100.0 %	0.0 %	100.0 %
Saint Ana Hospital	16	8	24
	66.7 %	33.3 %	100.0 %
Saint Marina Hospital	19	0	19
	100.0 %	0.0 %	100.0 %
Saint Cecilio Hospital	39	6	45
	86.7 %	13.3 %	100.0 %
Virgen de las Nieves Hospital	77	6	83
	92.7 %	7.3 %	100.0 %
Prevention and Control Tuberculosis Unit	35	0	35
	100.0 %	0.0 %	100.0 %
TOTAL	507	85	592
	85.6 %	14.4 %	100.0 %

The characteristics of the study population can be found in the Tables 2, 3 and 4. The majority of the cases presented with pulmonary or mixed TB, almost half were smear-positive, and more than one third presented with cavitation on chest x-ray. One third of the patients were over 50 years of age and almost two thirds were between 18 and 50 years old (64.7 %). More than 20 % of the cases lived alone, in a group, or were homeless, and 32.3 % were immigrants. More than half of the study population was diagnosed in the emergency department. Almost half (46.3 %) were smokers or ex-smokers, and around 20 % were alcoholics. HIV co-infection was present in 4.2 % of the population, although HIV status was unknown in 18.6 % of the cases. Almost 6 % of the TB cases were relapse. Four hundred and eleven (69.5 %) were diagnosed in either the emergency department or specialty clinics (Table 2).

**Table 2:** Clinical and epidemiological characteristics of tuberculosis cases

	Total		Reported	
	N = 592		N = 507	
Variables	n	%	n	%
Age (years)				
18-30	146	24.7	128	25,25
31-50	237	40	212	41,81
51-64	62	10.5	52	10,26
>65	121	20.4	92	18,15
Unknown	26	4.4	23	4,54
Sex				
Male	368	62.2	321	63,31
Female	214	36.1	179	35,31
Unknown	10	1.7	7	1,38
Employment				
Employed	205	34.6	174	34,32
Unemployed	184	31.1	169	33,33
Retired	125	21.1	94	18,54
Unknown/On disability	78	13.2	70	13,81
Living situation				
With family	423	71.5	371	73,18
Alone	42	7.1	33	6,51
In a group	64	10.8	53	10,45
Homeless	14	2.4	11	2,17
Incarcerated	10	1.7	8	1,58
Unknown	39	6.6	31	6,11
Center of diagnosis				
Emergency department	320	54.1	283	55,82
Primary care	113	19.1	94	18,54
Specialized center	91	15.4	76	14,99
Unknown or other	68	11.5	54	10,65
Smoking				
Non-smoker	318	53.7	272	53,65
Smoker	194	32.8	176	34,71
Ex-smoker	80	13.5	59	11,64
Alcohol Use				
Yes	116	19.6	104	20,51
No	467	78.9	395	77,91
Unknown	9	1.5	8	1,58
HIV status				
Positive	25	4.2	23	4,54
Negative	446	75.3	384	75,74
Not known by patient	110	18.6	91	17,95
Unknown	11	1.9	9	1,78
Previous tuberculosis treatment				
No	543	91.7	464	91,52
Yes	34	5.7	29	5,72
Unknown	15	2.5	14	2,76
Country of origin				
Spain	401	67.7	334	65,88
Other	191	32.3	173	34,12
Drug resistance				
No	579	97.8	496	97,83
Yes	13	2.2	11	2,17
Chest radiograph				
Abnormal with cavitation	208	35.1	191	37,67
Abnormal without cavitation	284	48	235	46,35
Normal	71	12	57	11,24
Unknown	29	4.9	24	4,73
Microbiology				
Smear-positive	287	48.5	260	51,28
Smear-negative and culture-positive	222	37.5	179	35,31
Smear-negative and culture-negative	67	11.3	55	10,85
Other	16	2.7	13	2,56
Tuberculosis involvement				
Pulmonary	405	68.4	362	71,40
Extrapulmonary	71	12	54	10,65
Mixed	44	7.4	36	7,10
Unknown	72	12.2	55	10,85

**Table 3:** Demographic characteristics of tuberculosis cases and factors associated with unreported cases

	Total	Reported		Unreported		Bivariate analysis		Multivariate analysis	
	N = 592	N = 507		N = 85		OR (95 % CI)	p-value	OR (95 % CI)	p-value
Variables		n	%	n	%				
Age (years)									
18-30	146	128	87.7	18	12.3	1.19 [0.62-2.27]	0.592		
31-50	237	212	89.5	25	10.5	Ref.	Ref.		
51-64	62	52	83.9	10	16.1	1.64 [0.71-3.56]	0.238		
>65	121	92	76	29	24	2.66 [1.48-4.84]	0.001		
Unknown	26	23	88.5	3	11.5	1.15 [0.25-3.66]	0.835		
Sex									
Male	368	321	87.2	47	12.8	Ref.	Ref.		
Female	214	179	83.6	35	16.4	1.34 [0.83-2.14]	0.235		
Unknown	10	7	70	3	30	3.00 [0.59-11.5]	0.165		
Employment									
Employed	205	174	84.9	31	15.1	1.54 [0.70-3.7]	0.296	1.75 (0.74-4.09)	0.197
Unemployed	184	169	91.8	15	8.2	0.77 [0.32-2.02]	0.581	0.88 (0.35-2.23)	0.801
Retired	125	94	75.2	31	24.8	2.84 [1.27-7.04]	0.01	3.04 (1.29-7.18)	0.011
Unknown/On disability	78	70	89.7	8	10.3	Ref.	Ref.		Ref.
Living situation									
With family	423	371	87.7	52	12.3	Ref.	Ref.		
Alone	42	33	78.6	9	21.4	1.96 [0.84-4.21]	0.116		
In a group	64	53	82.8	11	17.2	1.49 [0.70-2.96]	0.287		
Homeless	14	11	78.6	3	21.4	2.01 [0.42-6.81]	0.338		
Incarcerated	10	8	80	2	20	1.88 [0.25-7.95]	0.477		
Unknown	39	31	79.5	8	20.5	1.86 [0.76-4.12]	0.166		
Center of diagnosis									
Emergency Department.	320	283	88.4	37	11.6	Ref.	Ref.		
Primary care	113	94	83.2	19	16.8	1.55 [0.83-2.80]	0.163		
Specialty clinic	91	76	83.5	15	16.5	1.52 [0.77-2.87]	0.223		
Unknown or other	68	54	79.4	14	20.6	1.99 [0.98-3.88]	0.058		
Country of origin									
Spain	401	334	83.3	67	16.7	Ref.	Ref.		
Other	191	173	90.6	18	9.4	0.52 [0.29-0.89]	0.016

**Table 4:** Clinical characteristics of tuberculosis cases and factors associated with unreported cases

	Total	Reported		Unreported		Bivariate analysis		Multivariate analysis	
	N = 592	N = 507		N = 85		OR (95 % CI)	p-value	OR (95 % CI)	p-value
Variables		n	%	n	%				
Smoking									
Non-smoker	318	272	85.5	46	14.5	1.64 [0.94-3.00]	0.084		
Smoker	194	176	90.7	18	9.3	Ref.	Ref.		
Ex-smoker	80	59	73.8	21	26.3	3.46 [1.72-7.03]	0.001		
Alcohol use									
Yes	116	104	89.7	12	10.3	Ref.	Ref.		
No	467	395	84.6	72	15.4	1.56 [0.84-3.14]	0.162		
Unknown	9	8	88.9	1	11.1	1.20 [0.04-7.67]	0.877		
HIV status									
Positive	25	23	92	2	8	Ref.	Ref.		
Negative	446	384	86.1	62	13.9	1.74 [0.49-11.9]	0.435		
Not known by patient	110	91	82.7	19	17.3	2.25 [0.59-16.2]	0.264		
Unknown	11	9	81.8	2	18.2	2.48 [0.23-27.0]	0.431		
Previous tuberculosis treatment									
No	543	464	85.5	79	14.5	Ref.	Ref.		
Yes	34	29	85.3	5	14.7	1.04 [0.34-2.56]	0.942		
Unknown	15	14	93.3	1	6.7	0.48 [0.02-2.42]	0.436		
Drug resistance									
No	579	496	85.7	83	14.3	Ref.	Ref.		
Yes	13	11	84.6	2	15.4	1.15 [0.16-4.47]	0.861		
Chest radiograph									
Abnormal with cavitation	208	191	91.8	17	8.2	Ref.	Ref.		
Abnormal without cavitation	284	235	82.7	49	17.3	2.33 [1.32-4.29]	0.003		
Normal	71	57	80.3	14	19.7	2.75 [1.25-5.96]	0.012		
Unknown	29	24	82.8	5	17.2	2.37 [0.71-6.72]	0.149		
Microbiology									
Smear-positive	287	260	90.6	27	9.4	Ref.	Ref.		Ref.
Smear-negative and culture-positive	222	179	80.6	43	19.4	2.30 [1.38-3.91]	0.001	1.87 (1.07-3.28)	0.028
Smear-negative and culture-negative	67	55	82.1	12	17.9	2.11 [0.97-4.36]	0.059	1.59 (0.68-3.72)	0.280
Other	16	13	81.3	3	18.8	2.29 [0.48-7.80]	0.264	1.24 (0.30-5.06)	0.759
Tuberculosis involvement									
Pulmonary	405	362	89.4	43	10.6	Ref.	Ref.		Ref.
Extrapulmonary	71	54	76.1	17	23.9	2.65 [1.38-4.94]	0.004	2.07 (1.05-4.09)	0.035
Mixed	44	36	81.8	8	18.2	1.89 [0.77-4.18]	0.156	1.50 (0.63-3.53)	0.353
Unknown	72	55	76.4	17	23.6	2.61 [1.36-4.84]	0.005	2.01 (0.97-4.15)	0.059

*HIV*: Human immunodeficiency virus

On a bivariate level, the following variables were associated with unreported cases: age over 65 years, retirement, smoking history, immigrant status, normal or non-cavitary chest x-ray, smear-negative TB, and the presence of extrapulmonary TB. On a multivariate level, the following variables were associated with unreported TB: retirement (OR: 3.04, CI 1.29-7.18), smear- negative TB (OR: 1.87, CI 1.07-3.28) and the presence of extrapulmonary TB (OR: 2.07, CI 1.05-4.09) (Tables 3 and 4). The percentage of reported cases among the smear-positive cases was 9.4 % and 19.4 % (p = 0.001) among the rest of the study population. Smear-positive TB was no associated to under-reporting.

## Discussion

We found that 14.4 % of TB cases were not reported to a public health department and the proportion of unreported cases ranged between 0 and 45.2 % according to healthcare facility. It is notable that the five healthcare facilities that reported 100 % of their TB cases to the public health department employed a nurse case manager who acted as a liaison between the medical team and the infection control team, assisted with data collection, contact tracing, and case reporting to the public health department.

Previous studies in Europe have estimated the rate of unreported TB cases is over 20 % [7, 10, 11, 18, 19]. A rate of 27 % was described in central Italy [10], 38-49 % in the United Kingdom [7], and 80 % in Greece [11]. Studies from Spain estimate rates of unreported TB range from 20 % to 46 % [13–15, 19], but these percentages represent a limited geographical area (the Baleares Islands, Area 15 of Alicante, León and Asturias). This range is wide and may be due to the local TB organization.

Regarding the factors associated with unreported TB cases, studies have described high rates of unreported cases among older patients [10, 13], among those without microbiological confirmation [10], among patients with absence of cavitary lesions on chest x-ray [10, 13], and among non-immigrant patients [13]. Our study showed the same results on a bivariate level, but without statistical significance on a multivariate level for age, x-ray findings or country of origin. Retirement was associated with a higher risk of under-reporting, even independent of age. This has also been demonstrated in studies performed in other countries [20], which describe 25 % of unreported cases among patients over 60 years of age. This may be due to the higher rate of comorbidity conditions and multiple reasons for hospital admission, which could distract the provider that would diagnose and report the TB case.

We also found an association between unreported cases and extrapulmonary TB and smear-negative TB, for which the diagnosis may be delayed or without microbiological histology or culture. This was also described in many other studies [7, 10, 13, 20, 21], and maybe due to the fact that the provider think that transmission is lower among these cases. Nonetheless, reporting TB cases to the public health department is important to identify affected patients promptly thereby lowering transmission, to calculate an accurate incidence, and also to identify the TB index cases.

Smear-positive TB patients are more contagious and thus case reporting and contact tracing is crucial. Our study found that 9.4 % of smear-positive cases were not reported, which is actually lower than rates described in other studies [19].

The majority of the cases were diagnosed and reported from emergency departments and specialty clinics in our study as well as from other published studies [13, 15], and half of which were diagnosed in the emergency department. Case detection in the primary care setting is essential for early diagnosis and eventual disease control. We found that the diagnosis of TB in primary care centers is not associated with under-reporting (Table 3), which differs from one Spanish study [13]. However the percentage of TB cases diagnosed in primary centers is small and could represent an initial opportunity for diagnosis that was missed. This suggests disease control in the primary care setting may be weak and could be a target for strategies to improve TB diagnosis. Training programs for the diagnosis of TB targeting the general public and primary care providers should be implemented.

When TB is not diagnosed or unreported, an opportunity to prevent disease transmission is lost and the disease can spread. All patients with a concern for TB should be immediately evaluated and the diagnosis should be reported to the public health department without delay [5, 6]. This requires coordination between the hospital, the department of epidemiology, and the microbiology and pathology departments. For example, electronic reporting systems, in which case reports are sent electronically from local to centralized databases, have been implemented in other countries [22, 23].

Our study also has limitations that are inherent to retrospective studies because of missing information. However, a prospective study design could have led to a bias of high compliance and reporting. The large number of participating healthcare facilities in our study offers a good estimation of unreported TB cases, even with a retrospective design. Additionally, the number of patients who were not evaluated at a specialty clinic is low. The patients who were diagnosed and followed by primary care centers have microbiological data recorded in a microbiology registry compiled with data from specialty clinics, but no electronic medical record.

## Conclusions

It is important that TB Control Programs encourage thorough case reporting to improve disease control, contact tracing and accuracy of epidemiological data. This is particularly relevant for TB cases that are smear-negative, given the association with under-reporting. As seen from our study results, the help from a TB nurse case manager could improve the rate of under-reporting.

## Additional file

Additional file 1:
**Ethics.** (DOCX 10 kb)

## References

[1] WHO: Global Tuberculosis Report 2014. World Health Organization. WHO/HTM/TB 2014. Available: http://www.who.int/tb/publications/global_report/en/.

[2] European Centre for Disease Prevention and Control (ECDC)/World Health Organization Regional Office for Europe: Tuberculosis surveillance and monitoring in Europe 2014. Stockholm, 2014. Available: http://www.euro.who.int/__data/assets/pdf_file/0004/245326/Tuberculosis-surveillance-and-monitoring-in-Europe-2014.pdf?ua=1. Accessed 2014 Jun 10

[3] Orcau A, Caylà J, Martínez JA (2011). Present epidemiology of tuberculosis. Prevention and control programs. Enferm Infecc Microbiol Clin.

[4] Rodríguez E, Villarrubia S, Díaz O, Hernández G, Tello O. Situación de la tuberculosis en España. Casos de tuberculosis declarados a la Red Nacional de Vigilancia Epidemiológica en,2010. Bol Epidemiol Sem. 2012;20(3):26–41. Available: http://www.revista.isciii.es/index.php/bes/article/download/693/727 Accessed 2014 Jun 11.

[5] CDC (2009). Trends in tuberculosis United States, 2008. MMWR.

[6] CDC (2010). Decrease in Reported Tuberculosis Cases — United States, 2009. MMWR.

[7] Pillaye J, Clarke A (2003). An evaluation of completeness of tuberculosis notification in the United Kingdom. BMC Public Health.

[8] Theodoracopoulos P, Dimadi M, Constantopoulos SH (1992). Calculation of new cases of tuberculosis from the consumption of antituberculosis medications; comparison with notification rates. Respiration.

[9] Jelastopulu E, Alexopoulos EC, Venieri D, Tsiros G, Komninou G, Constantinidis TC, Chrysanthopoulos K. Substantial underreporting of tuberculosis in West Greece - implications for local and national surveillance. *EuroSurveill* 2009;14(11):pii = 19152. Available: http://www.eurosurveillance.org/ViewArticle.aspx?ArticleId=19152. 19317978

[10] Melosini L, Vetrano U, Dente FL, Cristofano M, Giraldi M, Gabbrielli L, et al. Evaluation of underreporting tuberculosis in Central Italy by means of record linkage. BMC Public Health. 2012;12:472. Available: http://www.biomedcentral.com/1471-2458/12/472. 10.1186/1471-2458-12-472 PMC3490729 22897910

[11] Lytras T, Spala G, Bonovas S, Panagiotopoulos T (2012). Evaluation of tuberculosis underreporting in Greece through comparison with anti-tuberculosis drug consumption. PlosOne.

[12] D’Ambrosio L, Centis R, Spanevello A, Migliori GB. Improving tuberculosis surveillance in Europe is key to controlling the disease. Euro Surveill 2010; 15 (11):pii = 19513. Available: http://www.eurosurveillance.org/ViewArticle.aspx?Articleld=19513. 20338148

[13] Giménez Duran J, Galmés Truyols AM, Guibert DH, Bonilla Vargas LA, Luque Fernández MA, Bosch CI, et al. Tuberculosis surveillance in the Balearic islands and characteristics of unreported cases from 2005 to 2007. Gac Sanit. 2011;25(1):84–6. 10.1016/j.gaceta.2010.09.016 21315493

[14] Calpe JL, Chiner E, Marín J, Martínez C, López MM, Sánchez E (2001). Tuberculosis notification from 1987 to 1999 for the public health area of the community of Valencia (Spain). Arch Bronconeumol.

[15] Múñiz-González F, Guerra-Laso J, García-García S, López-Veloso M, Raposo-García S, Carracedo-Falagán N, et al. Estimate of the real incidence of tuberculosis in the Leon Health Area: Application of the capture-recapture method to compare two information sources. Enferm Infecc Microbiol Clin. 2013;31(2):82–7. 10.1016/j.eimc.2012.06.009 22999799

[16] Altet Gómez MN, Alcaide Megías J (2006). Control and elimination of tuberculosis in Spain: Recommendations for the twenty-first century. An Pediatr (Barc).

[17] WHO: Treatment of tuberculosis guidelines, 4th ed. Geneva, World Health Organization, 2010 (WHO/HTM/STB/2009.420). Available: http://whqlibdoc.who.int/publications/2010/9789241547833_eng.pdf. Accessed 2013 april 2.

[18] Ködmön C, Hollo V, Huitric E, Amato-Gauci A, Manissero D. Multidrug- and extensively drug-resistant tuberculosis: a persistent problem in the European Union European Union and European Economic Area. *Euro Surveill* 2010; 15 (11):pii = 19519. Available: http://www.eurosurveillance.org/ViewArticle.aspx?Articleld=19519. 20338147

[19] Informe de la tuberculosis de Asturias (2007–2012). Available: http://www.asturias.es/Astursalud/Ficheros/AS_Salud%20Publica/AS_Vigilancia/Informes%20epidemiol%C3%B3gicos/2012_web.pdf.

[20] Baussano I, Bugiani M, Gregori D, van Hest R, Borracino A, Raso R, et al. Undetected burden of tuberculosis in a low-prevalence area. Int J Tuberc Lung Dis. 2006;10:415–21. 16602406

[21] Buiatti E, Acciai S, Ragni P, Tortoli E, Barbieri A, Cravedi B, et al. The quantification of tuberculosis disease in an Italian area and the estimation of underreporting by means of record linkage. Epidemiol Prev. 1998;22:237–41. 10052262

[22] Rolfhamre P, Jansson A, Arneborn M, Ekdahl K (2006). SmiNet-2. Description of an internet-based surveillance system for communicable diseases in Sweden. Euro Surveill.

[23] Krause G, Altmann D, Faensen D, Porten K, Benzler J, Pfoch T, et al. SurvNet electronic surveillance system for infectious disease outbreaks, Germany. Emerg Infect Dis. 2007;13:1548–55. 10.3201/eid1310.070253 PMC2851509 18258005

