# Supplementary material for: Factors associated with unreported tuberculosis cases in Spanish hospitals
Source: BMC Infect Dis. 2015 Jul 29;15:295. doi: 10.1186/s12879-015-1047-0 (PMC4518602; doi:10.1186/s12879-015-1047-0)
Supplement: Additional file 1: — Ethics. (DOCX 10 kb) [file 12879_2015_1047_MOESM1_ESM.docx]

The study was approved by the Independent Ethics Committees of the participating healthcare facilities: Saint Millan-Saint Pedro Hospital (Logroño), Tarrasa Health Consortium (Tarrasa), Carlos III Hospital (Madrid), Xeral-Calde Hospital (Lugo), Castellon General Hospital (Castellón), Saint Agustin Hospital (Avilés), Sierrallana Hospital (Torrelavega), Carlos Haya Hospital (Malaga), Asturias Central Hospital (Oviedo), Jaen Hospital (Jaen), Saint Boi Hospital (Sant Boi), Saint Ana Hospital (Motril), Saint Marina Hospital (Bilbao), Saint Cecilio Hospital (Granada), Virgen de las Nieves Hospital (Granada), Vall d'Hebrón Hospital (Barcelona), Mar Hospital (Barcelona).
